# Spatiotemporal analysis of *Escherichia coli* membrane permeabilization and uptake kinetics induced by a single microbubble cavitation event

**DOI:** 10.1038/s41598-025-26554-4

**Published:** 2025-11-28

**Authors:** Mitja Drab, Aleš Iglič, David Stopar, Žiga Pandur

**Affiliations:** 1 Faculty of Electrical Engineering, University of Ljubljana, Tržaška Cesta 25, 1000 Ljubljana, Slovenia; 2 Faculty of Mechanical Engineering, University of Ljubljana, Aškerčeva 6, 1000 Ljubljana, Slovenia; 3 Biotechnical Faculty, University of Ljubljana, Jamnikarjeva 101, 1000 Ljubljana, Slovenia

**Keywords:** Cavitation, Bacterial cells, Poration, Propidium iodide uptake, Goldman equation, Biological techniques, Biophysics, Engineering

## Abstract

A single cavitation microbubble can transiently disrupt the cellular membrane, providing a chemical-free, targeted drug delivery mechanism. Here, we investigate the spatiotemporal dynamics of membrane permeabilization in an *Escherichia coli* (*E. coli*) monolayer exposed to a single cavitation event. Using high-resolution fluorescence microscopy and propidium iodide (PI) uptake as a marker of membrane disruption, we tracked the response of 5565 individual cells around the center of the cavitation event over timescales from microseconds to minutes and spatial scales from 1 to 165 µm. PI uptake rates exhibited a strong spatial dependence, with cells closer to the cavitation center showing rapid and extensive permeabilization. A modified Goldman equation describing PI concentrations inside and outside the cells was used and related to the spatiotemporal measurements of fluorescence intensity. The model accurately captured the first-order PI uptake kinetics, which resulted in saturated fluorescence intensity profiles. Additionally, the model predicted an exponential decay of permeability post-cavitation, implicitly suggesting that pore-resealing dynamics were taking place. Membrane permeability decreased with distance as 1/r, with a characteristic decay time of approximately 3.4 min. Our model thus predicts cell damage induced by a single cavitation event in both space and time. In the present case, where the cavitation bubble reached maximum radius of 29.7 µm, we found that at a distance of 11 µm from the cavitation center, ~ 50% of cell membranes are damaged and permeable to PI, but a pore-resealing mechanism reduces this damage to ~ 1% after 10 min. Our results are consistent with existing sonoporation studies and offer novel insights for optimizing cavitation-assisted drug delivery and biofilm disruption strategies.

**Supplementary Information:**

The online version contains supplementary material available at 10.1038/s41598-025-26554-4.

## Introduction

Cavitation-induced drug delivery system (i.e. sonoporation) is a promising strategy for enhancing cell membrane permeability in therapeutic and biotechnological applications^1,2^. The rapid collapse of microbubbles generates transient, high-intensity mechanical stresses that temporarily permeabilize biological membranes, enabling the targeted delivery of antimicrobial agents, genetic material, and other therapeutic compounds into cells^3–9^. Sonoporation relies on short, intense tensile forces to form transient membrane pores, offering advantages over traditional electroporation by producing fewer but larger pores and minimizing collateral damage when precisely controlled^10–13^. Sonoporation is particularly attractive for delivering macromolecules that cannot easily cross cellular membranes. Unlike stable protein-mediated pores, which offer selectivity but require complex regulation, transient pores induced by mechanical forces enable rapid influx of large molecules, albeit with reduced control^11^. Cavitation mediated permeabilization of bacterial cells is used for DNA transformation and gene delivery, nanoparticle delivery or enhanced antibiotic uptake^14–16^. The effect of cavitation on bacteria is usually studied using a statistical multi-bubble approach. Here, the neighboring bubbles can interact non-linearly, either increasing or decreasing the effect on bacteria. To better understand the cavitation effect on bacteria, it is necessary to scale down to a single-bubble level. In addition, the evolution of bacterial damage over space and time would allow one to identify cause-effect relationships that would help predict and optimize sonoporation-based cavitation delivery systems. While quasi-static techniques such as micropipette aspiration, flow cytometry, and atomic force microscopy have been used to assess poration thresholds, they lack the temporal resolution to capture the rapid, high strain-rate conditions induced by cavitation. The rapid collapse of a single cavitation bubble near a surface-attached bacterial cell imposes mechanical forces, hydrodynamic pressure and shear stress on sub-microsecond time scales. Recent work by Pandur et al. identified mechanical load thresholds for bacterial cell detachment and death under such conditions^17^. However, the molecular transport dynamics through mechanically induced pores remain poorly understood. Gram-negative bacteria such as *Escherichia coli* (*E. coli*) possess a complex dual-membrane structure that acts as a significant barrier to the transport of charged molecules^18^. Propidium iodide (PI) is a small, hydrophilic, cationic dye (M_w_ = 668 Da), that cannot cross the intact bacterial membranes due to its positive charge. In compromised membranes, PI crosses the membrane and intercalates with DNA, where its quantum yield increases significantly, making it a widely used indicator of cell membrane damage^19^.

In this study, we employed a single microbubble cavitation event, precisely positioned and triggered near a surface-attached layer of *E. coli* cells and visualized it using ultra-high-speed imaging. The timing and location of the microbubble event were precisely regulated on the µs and µm scale. The resulting effects of a single microbubble cavitation event on the permeability of individual bacterial membranes were assessed through PI staining and time-resolved fluorescence microscopy. The results showed that PI uptake decreased radially symmetrically from the microbubble’s cavitation site, suggesting a similar dependence of damage inflicted on cells. Based on the experimental results, we modeled the uptake kinetics of PI using a modified Goldman equation. We hypothesized that PI concentration inside the cells is in direct proportion to the measured fluorescence signal. This allowed us to derive an analytical expression for the spatiotemporal dependence of the fluorescence signal, which showed good agreement with experiments. Furthermore, our model predicted a spatiotemporal dependence of the permeability of damaged cells, which was greatest at the time of the cavitation event and then decreased exponentially with a characteristic time of 3.4 min. We attributed this decrease of PI uptake on a spontaneous pore-resealing mechanism. Finally, our model allowed us to estimate the damaged area of nearby cells as a function of distance and time from the center of the cavitation event—values, that are experimentally inaccessible. Our combined experimental and numerical approach offers a mechanistic framework for quantifying membrane poration induced by a single microscale cavitation event. It also provides valuable spatiotemporal insights relevant to applications in drug delivery, microbial control, and mechanobiology.

## Materials and methods

### Single microbubble generation

The experimental setup is described thoroughly in Pandur et al.^17^. Briefly, a combination of optical tweezers (Aresis d.o.o., Ljubljana, Slovenia) and high-voltage electric discharge was used to generate micrometer-sized cavities. A laser was focused on a micrometer-sized magnetic bead (ProMag 3 Series—3 µm particle diameter, Bangs Laboratories, Indiana, United States of America) to create a stable micrometer-sized vapor microbubble, which served as the initial bubble nucleus. A tension wave generated by a spark discharge was then used to promote nucleus expansion and subsequent violent collapse—all on a micrometer scale. The entire setup was mounted on a Nikon Eclipse Ti-U inverted microscope (Nikon, Shinagawa, Tokyo).

### High-speed image recording

To visualize the microbubble collapse, the microscope was coupled with an ultra-high-speed recording camera (Kirana 7 M; Specialised Imaging, Pitstone, United Kingdom), and illumination was provided by a pulsed laser diode (Cavilux Smart, 640 nm; Cavitar Tampere, Finland). For image acquisition, a 60 × water immersion objective (CFI Apo NIR 60X W; Nikon, Shinagawa, Tokyo) was used. Image sequences were captured at 5,000,000 frames per second, with a shutter speed of 0.14 µs and a resolution of 924 × 768 pixels. Camera acquisition was synchronized with the microbubble collapse using a photodiode trigger that detected the spark discharge event.

### Growth and preparation of bacteria

The preparation of the bacterial strain is described in detail by Pandur et al. 2023^17^. Briefly, an overnight culture of *E. coli* MG1655 was used to attach the cells to poly-L-lysine (PLL)-coated cover glass. To detect membrane permeability of the cells, the membrane-impermeable fluorescent dye propidium iodide (PI) was added to the experimental channel at a final concentration of 60 nM. The density of bacterial cells on cover glass was 0.28 cells/µm^2^.

### Fluorescence microscopy

To visualize the bacteria, an EMCCD camera (iXon 888 Ultra; Andor, Oxford Instruments, Abingdon, United Kingdom) was used in both brightfield and epifluorescence modes (excitation: 550/10 nm; emission: 640/40 nm). The exposure time was 200 ms, gain was set to 20, and the image size was 1024 × 1024 pixels (corresponding to a 223.5 × 223.5 µm field of view). For time-resolved microscopy, multi-dimensional acquisition mode was used, with time-lapse imaging set to 0.5 s intervals and a total of 1300 frames acquired (total acquisition time approx. 10 min). Brightfield images were captured both before the microbubble collapse and after the time-resolved fluorescence acquisition.

### Experimental synchronization

Synchronization of the experimental setup was controlled via a LabView (National Instruments, Austin, USA) script that triggered the consecutive steps of the experiment. The script synchronized the optically triggered laser pulse to the high-voltage spark discharge, and the motorized rotation of the microscope mirror to enable sequential recording on the two cameras (fluorescence and ultra-fast). The procedure was executed as follows: (i) The microbead was manually positioned at the image center under low-intensity optical trapping with the optical tweezer system. (ii) Fluorescence image acquisition was initiated. (iii) The LabView script was executed, which first rotated the microscope mirror to project the image onto the ultra-fast camera. It then triggered the high-intensity optical tweezer laser pulse to generate a micrometer-sized nucleation bubble. Immediately afterward, the high-voltage discharge was initiated, producing a shockwave that propagated toward the nucleation bubble and induced cavitation. The ultra-fast camera was synchronized via a photodiode signal detecting the spark discharge, which triggered recording of the ultra-fast camera. After the bubble collapse, the mirror was rotated back to direct imaging to the fluorescence camera (with a delay of ~ 3 s from cavitation to fluorescence imaging). Finally, fluorescence images were acquired at 0.5 s intervals.

### Image analysis

Microscopic image analysis was performed using Fiji software (version 1.53f51)^20^. First, the series of fluorescence images was aligned to the final frame to eliminate any passive sample movement during acquisition. Additionally, the initial frame of the sequence was subtracted from all subsequent frames to reduce background intensity. Since magnetic beads exhibited autofluorescence under the given imaging conditions, they were removed from the images by thresholding circular particles with an area greater than 5 µm^2^ and fluorescence intensity above 15,000 arbitrary units (AU). The brightfield image acquired at the end of the experiment was then used to create a cell mask. Using the ROI Manager, individual cells were selected, cropped, and their fluorescence intensity was measured across the entire time-resolved image stack. Selection and analysis were performed for individual cells located within a field of view corresponding to 6 × the maximum bubble radius. Fluorescence intensity and position of individual cells were exported as a text file for further analysis. The analysis was automated using a custom-written ImageJ macro.

The positions of individual cells were used to calculate their distance (${l}_{cell}$) from the site of microbubble collapse, defined as the cavitation center (CC) at the center of the image. This distance was computed as the vector magnitude from the x and y coordinates of each cell relative to the CC:

1 $$\:{l}_{cell}=\sqrt{{(x-center)}^{2}+{(y-center)}^{2}}$$

**Fig. 1:**
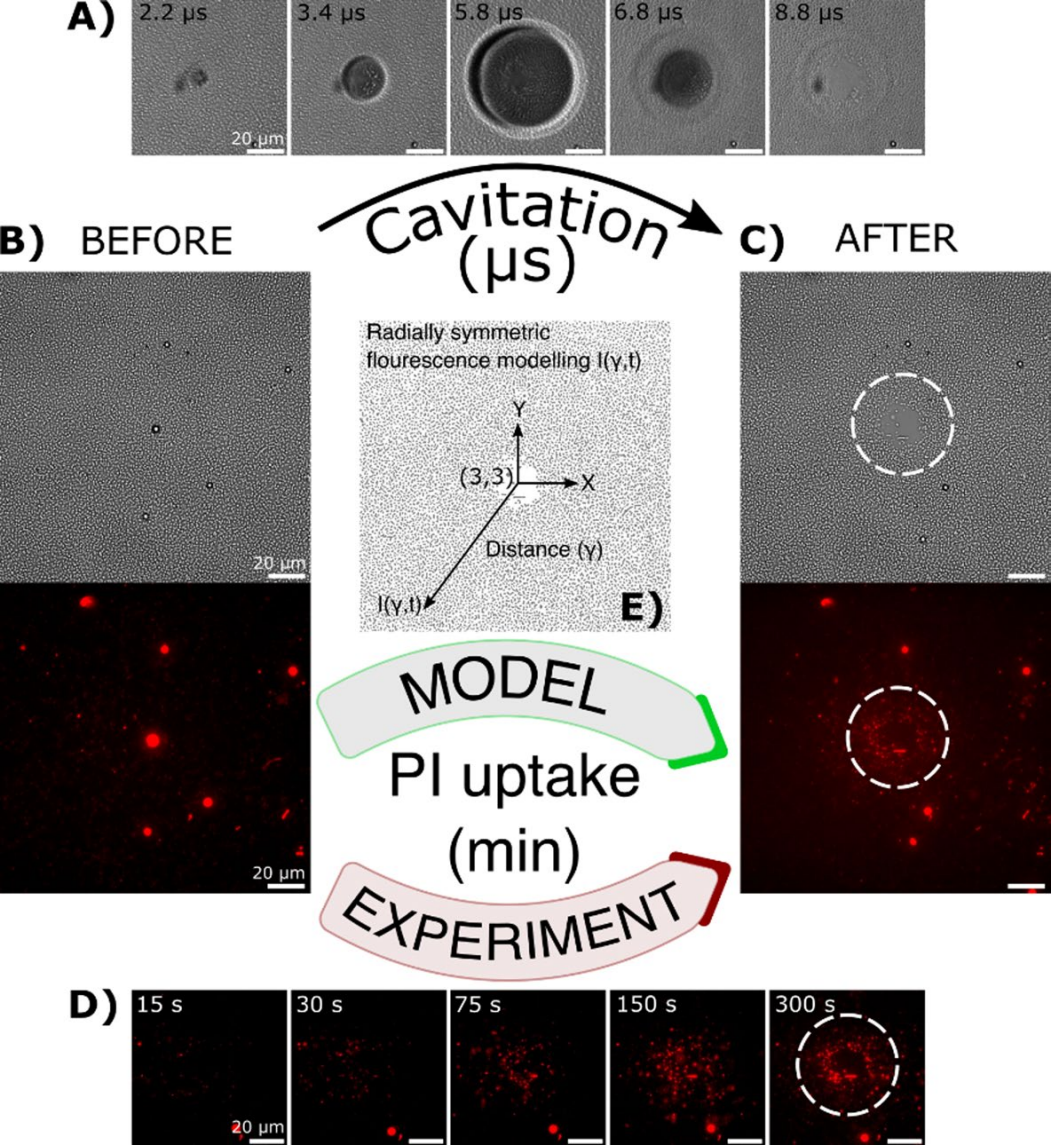
Experimental and numerical setup for spatiotemporal assessment of a representative single microbubble cavitation on bacterial cells. (**A**) Cavitation dynamics were captured using an ultra-high-speed imaging system. From left to right: following the passage of a tension wave, the bubble begins to expand (2.2 and 3.4 µs), reaching its maximum radius at ${\text{t}}_{{{\text{max}}}} = 5.8$ µs and then collapses (6.8 and 8.8 µs). (**B**) A uniform distribution of bacterial cells is shown in the brightfield image. A circular object at the center is a magnetic microbead that was used to generate a nucleation bubble. (**C**) The effect of cavitation on bacterial cells. In the post-event brightfield image, an annular region of detached cells is visible near the center. The fluorescence image (acquired ~ 10 min after cavitation) reveals cells stained with PI, indicating membrane disruption. Stained cells are pseudo-colored red. (**D**) Fluorescence over time, capturing PI uptake dynamics. Cells closer to the cavitation center (CC) fluoresce earlier than those farther away. White dotted circles indicate the projected maximum bubble radius. Scale bar = 20 µm. (**E**) The numerical domain corresponding to the experimental setup. The 2D Cartesian grid originates from the lower-left corner, with the cavitation center located at the non-dimensional coordinates (3,3), defining the fluorescence intensity function ${\text{I}}\left( {{\upgamma },{\uptau }} \right)$.

Note that in the 2D coordinate system, the CC is at point ($\gamma$ = 3, $\gamma$ = 3) (see Fig. 1). Here, $\gamma$ represents the non-dimensional distance of the cell from the CC, normalized by the microbubble’s maximum radius (${R}_{\text{max}}$):

2 $$\:\gamma\:=\frac{{l}_{\text{c}\text{e}\text{l}\text{l}}}{{R}_{\text{m}\text{a}\text{x}}}$$

The measured projected maximum radius (${R}_{\text{max}}$) of the microbubble were performed from ultra-high-speed microscopic images. The microbubble size was determined by manually detecting the bubble edge (dark edge on bright background) on the micrograph at fully expanded bubble and measuring its maximal diametral distance. Maximum bubble radius in given case was 29.7 µm.

### Statistical analysis

The statistical analysis of time-dependent fluorescence was performed in Python, using the curve_fit function from SciPy, which applies non-linear least squares optimization with the Levenberg Marquardt algorithm. The initial parameter estimates were provided through ${p}_{0}$, set to the maximum of the data and $0.01$, serving as starting points for the optimization. Additional fitting was performed in Mathematica 12.0 with the function NonlinearModelFit. Here, the ${R}^{2}$, RMSE, and residuals were calculated according to the following method: The coefficient of determination ${R}^{2}$ is calculated as ${R}^{2}=1-\left(\frac{S{S}_{res}}{S{S}_{tot}}\right)$, where $S{S}_{res}={\sum }{\left({y}_{i}-{Y}_{i}\right)}^{2}$ is the residual sum of squares, ${y}_{i}$ are the observed values, and ${Y}_{i}$ are the predicted values from the model. $S{S}_{tot}={\sum }{\left({y}_{i}-\overline{{y}_{i}}\right)}^{2}$ is the total sum of squares, with $\overline{{y}_{i}}$ being the mean of the observed values. The residuals are the differences between observed and predicted values, residuals = ${y}_{i}-{Y}_{i}$ for each data point. By plotting these residuals against the independent variable or against the fitted values, one can visually assess whether there is any systematic pattern left unexplained by the model. The ideal case is where the residuals are scattered homogeneously around the axis 0, which is approximately the case in our analysis. The root mean squared error (RMSE) is calculated as RMSE = $\sqrt{\overline{{\left({y}_{i}-{Y}_{i}\right)}^{2}}}$, which is the square root of the average squared residual. RMSE represents the typical size of the error in the same units as the dependent variable, making it easy to interpret relative to the scale of the data.

## Results

### Experimental results

To induce high-frequency mechanical shear stress perturbations in *E. coli* cells, the collapse of a single micrometer-scale cavitation bubble was employed. The cavitation event was initiated by laser-induced generation of a nucleation bubble on a magnetic microbead, followed by a high-voltage spark discharge that produced a shockwave in the surrounding medium, triggering the microbubble’s collapse. Solely laser irradiation of microbeads or shockwave generation didn’t have effect of PI intake of bacterial cells as observed before^17^.

The cavitation dynamics near the surface-attached layer of *E. coli* were captured using high-speed imaging at 5 million frames per second (Mfps), synchronized with a pulsed laser illumination system (Fig. 1A; Supplementary Video 1). Prior to the cavitation event (Fig. 1B), bacterial cells appeared evenly distributed throughout the field of view (FOV). The circular bright features in the brightfield image correspond to magnetic beads, which served as nucleation sites for the laser-generated bubble. The beads also auto fluoresce in the spectrum we’ve used for PI stain detection; therefore they are also visible under the fluorescence micrographs as a big circular objects (Fig. 1, fluorescent micrographs). The magnetic bead was positioned at the center of the image. After the shockwave propagated through the experimental chamber, the nucleation bubble expanded to a maximum radius of ${R}_{\text{max}}=29.7$ µm after 5.8 µs, followed by the bubble collapse phase. The entire cavitation event was completed within approximately 12 µs. Following the collapse, a clearly visible area of detached cells was observed in the bacterial layer, indicating the impact of the cavitation event (Fig. 1C).

To evaluate bacterial membrane damage, the membrane-impermeant, DNA-binding dye propidium iodide (PI) was added to the medium prior to the experiments. Time-lapse fluorescence microscopy was used to monitor the dynamics of PI uptake. Before the single cavitation event, background PI fluorescence was low, and brightfield images showed a uniform distribution of attached bacteria throughout the field of view (Fig. 1B). Following the bubble collapse, the number of cells with compromised membranes increased radially over the total observation time of $\tau = 600\text{ s}$. This pronounced increase in PI-positive cells correlated closely with the maximum size of the fully expanded microbubble (Fig. 1C and D).

### Fluorescence intensity saturation follows a first-order kinetics

**Fig. 2:**
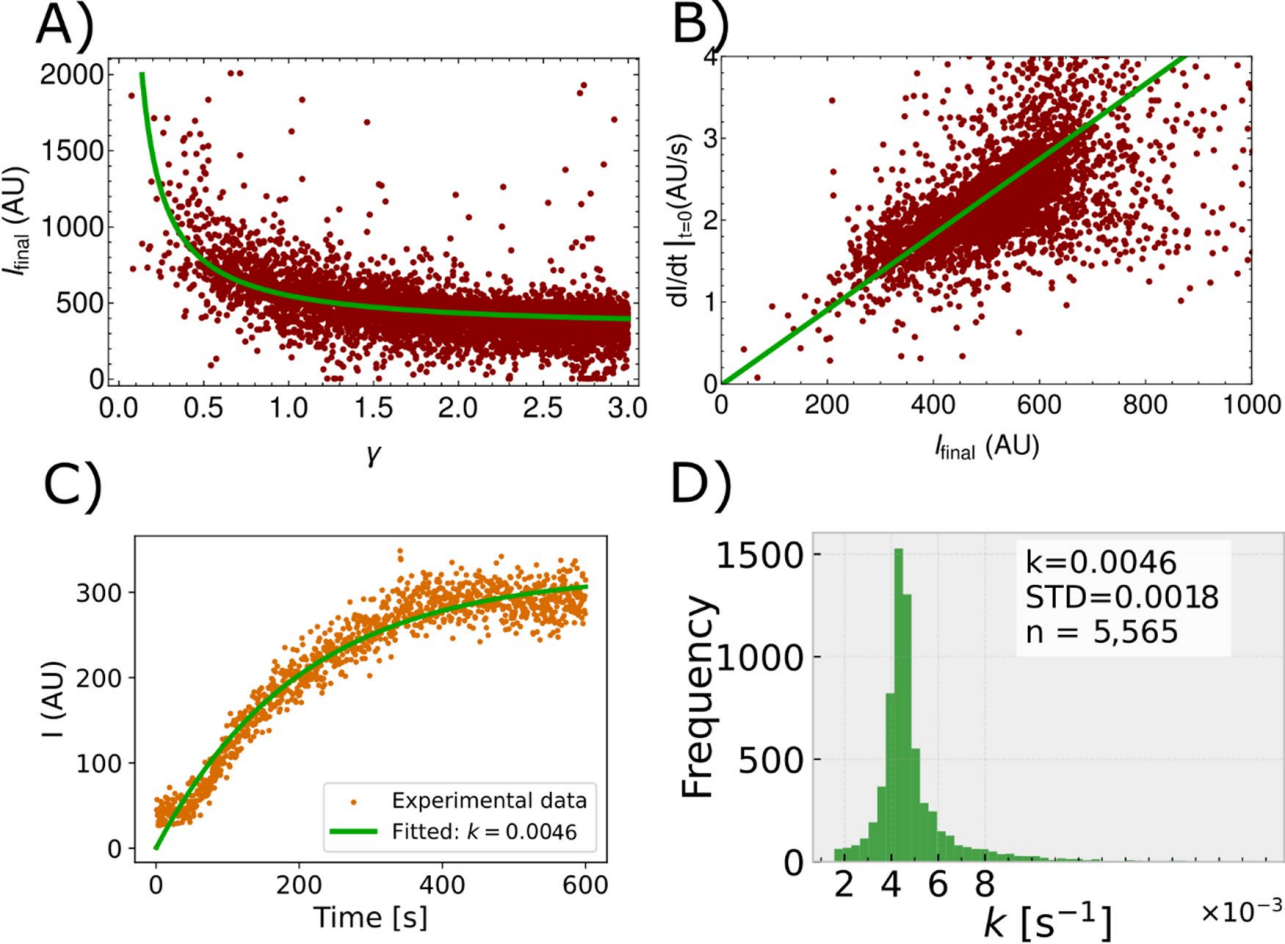
(**A**) Final cell fluorescence ${\text{I}}_{{{\text{final}}}} \left( {\upgamma } \right)$ decreases with distance from the cavitation center (CC) for 5,565 cells (red dots). The nonlinear dependency (${\text{R}}^{2} = 0.9$) suggests that ${\text{I}}_{{{\text{final}}}} \left( {\upgamma } \right)$ is correlated with the impact of the cavitation bubble and follows an inverse relationship (fitted ${\text{I}}_{{{\text{final}}}} \left( {\upgamma } \right) = 321 + \frac{228}{{\upgamma }}$ (green line)). (**B**) The linear relation between initial fluorescence rate ${\text{I}}\left( {{\upgamma },{\uptau }} \right)$ and final fluorescence intensity ${\text{I}}_{{{\text{final}}}}$ measured from experiments (red dots). The green line has the slope of the time constant ${\text{k}} = 0.0046{ }$ s^−1^ (${\text{R}}^{2} = 0.87$) obtained by fitting the saturation curves. The increase suggests a first-order kinetics mechanism; the cells that uptake PI faster also end up giving off a larger intensity signal ${\text{I}}_{{{\text{final}}}}$ at the end of observation time ${\text{t}} = 600$ s. (**C**) Experimental results for time dependence of ${\text{I}}\left( {{\upgamma },{\uptau }} \right)$ for a single cell (orange dots) show a saturation dynamic which is fitted well by a saturation curve (Eq. (7)) (${\text{R}}^{2} = 0.95$). The characteristic time constant is given by ${\uptau } = 1/{\text{k}}$ as described in the text. (**D**) The characteristic uptake constant ${\text{k}}$ for all relevant cells shows a distribution around a mean value of ${\text{k}} = 0.0046 \pm 0.0018{ }$ s^−1^. Values of three standard deviations away from mean were not taken into an account.

The fluorescence intensity was considered only for cells within the distance of $\upgamma \le 3$ from the CC (Eq. 2). The fluorescence intensity $I$ for all considered cells ($N=\text{5,565},\upgamma \le 3$) is a function of both space (distance from the CC) and time. The final fluorescence intensity, defined as ${I}_{\text{final}}=I(\upgamma ,\tau )$, varied with normalized distance from the CC, $\upgamma$. Plotting ${I}_{\text{final}}$ against $\upgamma$ showed a nonlinear decrease where cells closer to the CC exhibited higher ${I}_{\text{final}}$ values (Fig. 2A). The initial rate of fluorescence increase was, on average, faster in cells that ultimately reached higher ${I}_{\text{final}}$ values (Fig. 2B), suggesting that PI uptake saturated over time to values that were correlated with its initial rate of increase. This was confirmed by individual cell analysis, where $I(\text{t})$ saturated to a constant value by $\tau$ (Fig. 2C). The uptake mechanism therefore followed a first-order kinetics (Fig. 2B):

3 $$\frac{\partial I}{{\partial t}}|_{t = 0} = kI_{{{\text{final}}}}$$

**Fig. 3:**
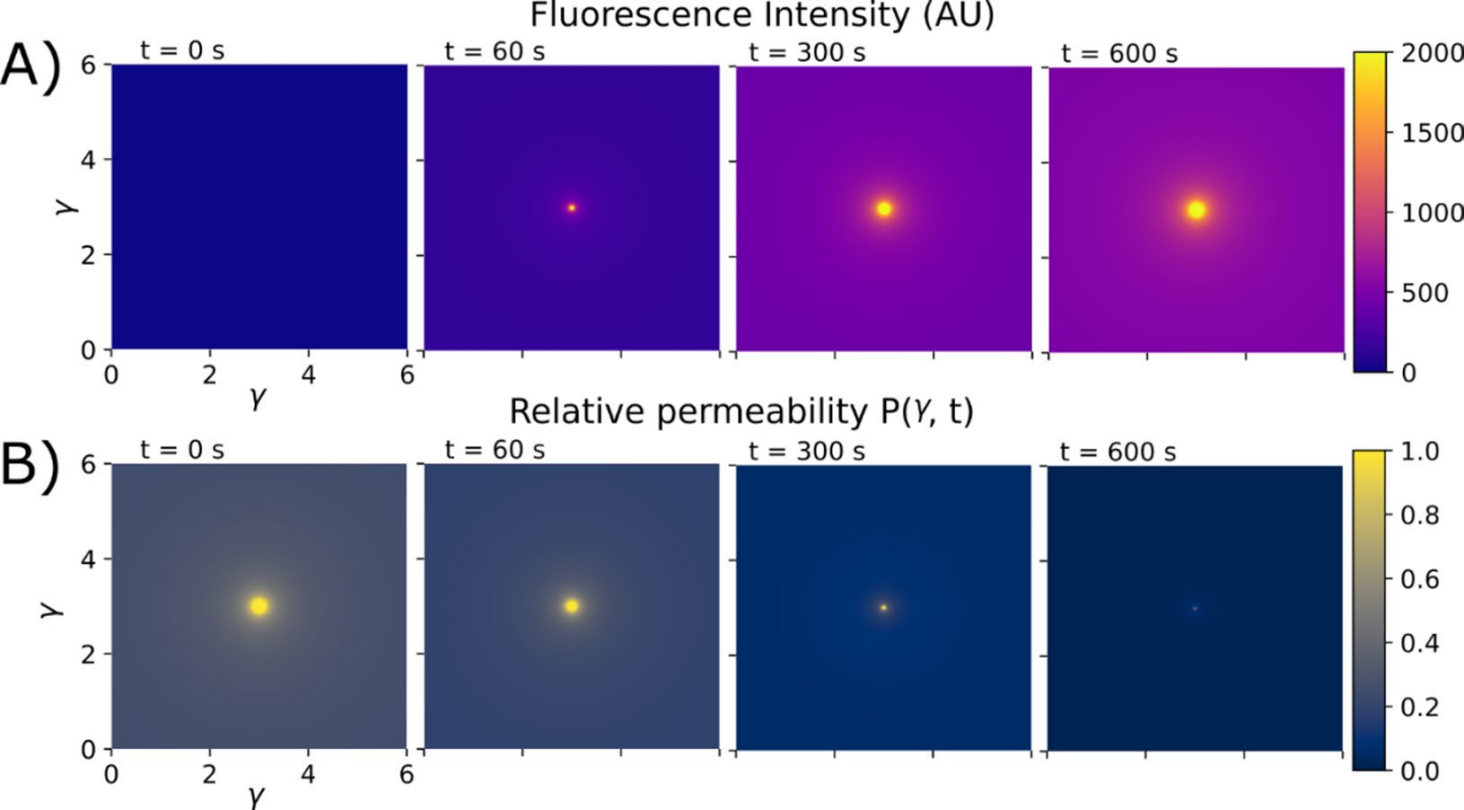
Results of the numerical model presented in a 2D plane for distance ${\upgamma } = 3$ from the CC. (**A**) Spatiotemporal distribution of fluorescence intensity ${\text{I}}\left( {{\upgamma },{\uptau }} \right)$ shows a radially dependent saturation (Eq. (7)). (**B**) Spatiotemporal relative permeability ${\text{P}}\left( {{\upgamma },{\uptau }} \right)$ shows an exponentially decreasing value over the time of observation (Eq. (9)). Note that here we plot dimensionless permeability which is normalized with respect to ${\text{p}}_{0}$. After cavitation collapse at (γ = 3, γ = 3), the highest permeability of the cell is expected near the cavitation center (CC) and decreases with distance and time.

After analyzing all cells within the affected region we determined an average time constant for PI uptake of $k = 0.0046 \pm 0.0018$ s^−1^, corresponding to a characteristic saturation time of $\tau = \frac{1}{k} = 217.39 \pm 84.91$ s (Fig. 2D). Equation (3) implies that $I\left( t \right)$ evolves as an exponential saturation curve (Fig. 2C):

4 $$I\left( t \right) = I_{{{\text{final}}}} \left( {1 - e^{ - kt} } \right)$$

where $I_{{{\text{final}}}}$ depends on the distance from the cavitation $I_{{{\text{final}}}} \left( \gamma \right) \propto 1/\gamma$ (Fig. 2A). The $1/\gamma$ decay is expected if we consider that energy released by a microbubble collapse spreads symmetrically over a 2D surface of the micrograph. The work $\delta W_{c}$ that the bubble collapse front does on the surrounding cell membranes is

5 $$\delta W_{c} = p\delta A$$

where $\delta A$ is the area of the annulus of width $\delta \gamma$ at distance $\gamma$ from the CC, over which the pressure $p$ acts on surface of cells. The area of the annulus is $\delta A = 2\pi \gamma \delta \gamma$, so its pressure changes as $p = \delta W_{c} /2\pi \gamma \delta \gamma$, decreasing with distance as $p\left( \gamma \right) \propto 1/\gamma$, like $I_{{{\text{final}}}} \left( \gamma \right)$. We find that $I_{{{\text{final}}}} \left( \gamma \right)$ can be fitted well by

6 $$I_{{{\text{final}}}} \left( \gamma \right) = 321 + \frac{228}{\gamma }$$

in arbitrary units of intensity (AU) (Fig. 2A). $I\left( {{\upgamma },\tau } \right)$ can therefore be written as (Fig. 3A)

7 $$I\left( {\gamma ,t} \right) = I_{{{\text{final}}}} \left( \gamma \right)\left( {1 - e^{ - kt} } \right)$$

Characteristic permeability of the cell membranes to PI, $p_{0}$, is estimated from the diffusion constant $D$ of PI ($10^{ - 12}$ $m^{2} /s$)^21^ and $L$, the length of a transmembrane *E. coli* pore (50 nm)^22^, so that

8 $$p_{0} = D/L = 2 \cdot 10^{ - 5} {\text{m/s}}p_{0} = D/L = 2 \cdot 10^{ - 5} {\text{m/s}}$$

Not all cells are equally permeable to PI, so the permeability of cells is a function of space and time $P\left( {\gamma ,t} \right)$. Furthermore, it is reasonable to assume that permeability is in direct proportion to PI uptake $\frac{\partial I}{{\partial t}}$ and hence the time derivative of $I\left( {\gamma ,t} \right)$ (Eq. 6):

9 $$P\left( {\gamma ,t} \right) = k_{1} \frac{\partial I}{{\partial t}} = kk_{1} I_{{{\text{final}}}} \left( \gamma \right)e^{ - kt}$$

Here, $k_{1}$ is a parameter with units m/AU that relates $P\left( {\gamma ,t} \right)$ to $\frac{\partial I}{{\partial t}}$. We observe that permeability is largest at the time of the microbubble collapse at $t = 0$, but then decreases with characteristic time constant $k$ (Fig. 3B). The exponential decay of PI uptake reflects pore closure over time (Fig. 3B). Cell membrane permeability is, on the other hand, connected to the density of pores in all cells’ membranes contained within the area of the annulus is $\delta A$ per unit of area $n$^23^, so that

10 $$\frac{P}{{p_{0} }} = n\pi r_{p}^{2} .$$

**Fig. 4:**
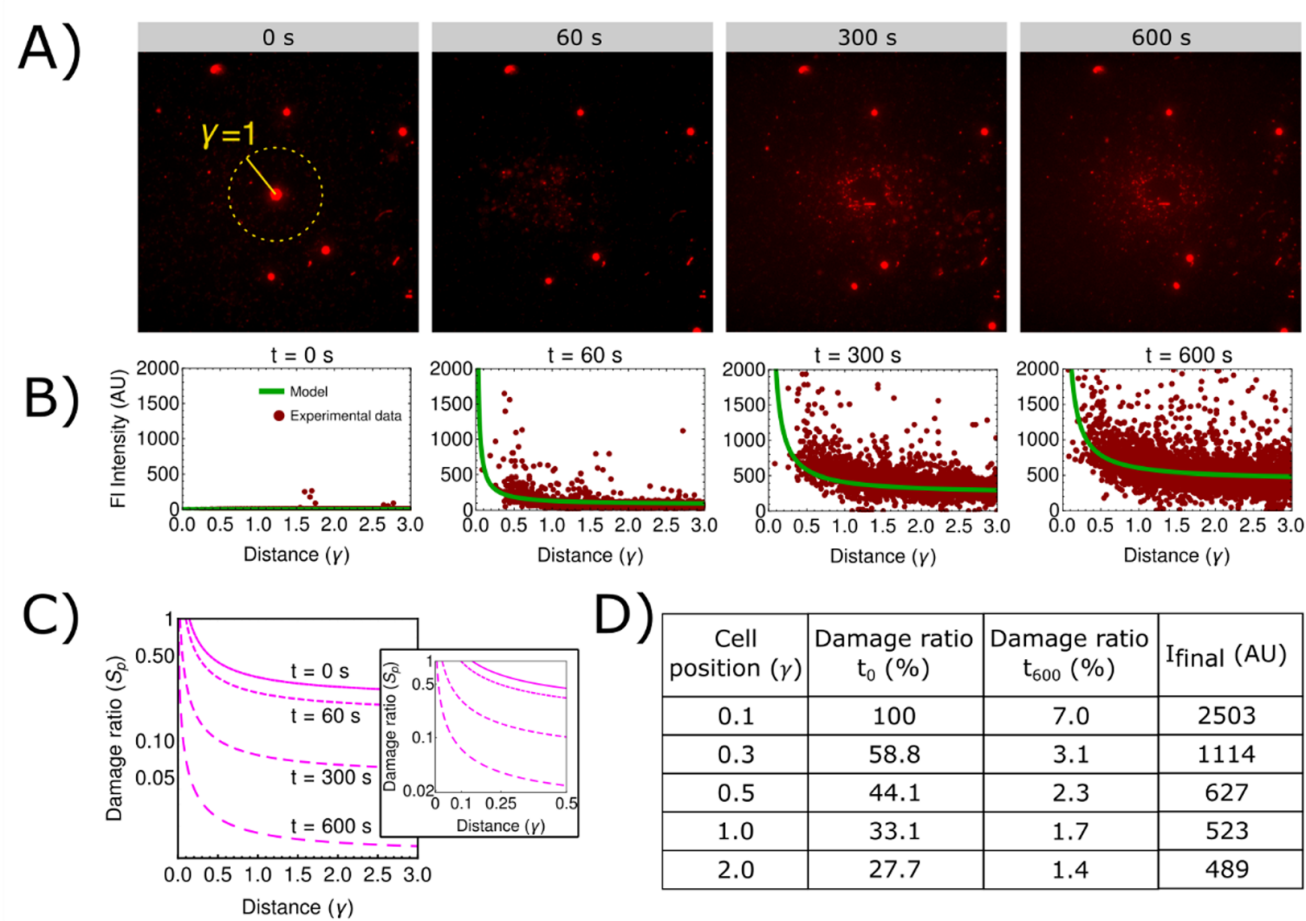
Comparison of experimental and calculated fluorescence intensity ${\text{I}}\left( {{\upgamma },{\text{t}}} \right)$ results. (**A**) Experiment micrographs show that the fluorescence intensity signal increases radially from the center and saturates over time of observation ${\uptau }$. The left panel shows the distance from the CC where ${\upgamma } = 1$. (**B**) Spatiotemporal fluorescence intensity ${\text{I}}\left( {{\upgamma },{\text{t}}} \right)$ results obtained by experiments (red dots) and modeling (green line, given by Eq. (7)) show good agreement (${\text{R}}^{2} = 0.9$). In the beginning (left panel, ${\text{t}} = 0$ s), no fluorescence signal is recorded from cells, but only from calibration beads, shown by several points. Over time, the fluorescence signal increases more strongly in cells located closer to the cavitation center (CC), and less so in those farther away (middle two panels). At the final time of observation (right panel, ${\text{t}} = 600$ s), the intensity profile is given by ${\text{I}}_{{{\text{final}}}} \left( {\gamma ,\tau } \right){\text{I}}_{{{\text{final}}}} \left( {\gamma ,\tau } \right)$ (Eq. (6)). (**C**) Results of spatiotemporal distribution of the damage ratio S_p_ (γ, τ) from numerical analysis (Eq. (11)). The ${\text{S}}_{{\text{p}}}$ was the highest right after bubble collapse (${\text{t}}_{{0}} = 0$ s) and then decreased with distance as well as time. (**D**) Model estimations of the damage ratio at the beginning (${\text{t}}_{{0}} = 0$ s) and at the end ${\text{t}}_{{{600}}} = 600$ s, and the maximum ${\text{I}}_{{{\text{final}}}}$ at various bubble-cell distances ${\upgamma }$.

Here, $r_{p}$ is the radius of an average pore. The area fraction of pores on a single *E. coli* cell is $S_{p} = n\pi r_{p}^{2}$, but $n$ is related to permeability by Eqs. (10) and (9). This results in an estimation of the damage fraction (Fig. 4C):

11 $$S_{p} \left( {\gamma ,t} \right) = \frac{{kk_{1} }}{{p_{0} }}\left( {a + \frac{b}{\gamma }} \right)e^{ - t/T} .$$

Here, constants $a = 321$ AU and $b = 228$ AU have units of fluorescence intensity and are obtained by fitting $I_{{{\text{final}}}}$. The parameter $k_{1} = 0.011$ m/AU was chosen so that cells that surpassed 2500 AU were considered totally damaged (100%). Additionally, we hypothesized that PI concentration inside the cells is in direct proportion to the measured fluorescence signal. We can then relate $I_{{{\text{final}}}} \left( \gamma \right)$ with $c_{2} \left( {\gamma ,t} \right)$ with a linear relationship $I_{{{\text{final}}}} \left( \gamma \right) = k_{2} c_{2} \left( {\gamma ,t} \right)$, where $k_{2} = 1.09 \cdot 10^{7}$ AU $m^{3}$/mol to get full spatiotemporal concentration and fluorescence intensity profiles (Supplementary video 3).

### Modeling PI uptake by a modified Goldman equation

To validate our data analysis, we developed a numerical model of propidium iodide (PI) uptake by *E. coli* cells using a modified Goldman equation. Owing to the radial symmetry of the cavitation bubble collapse, the dynamics of PI uptake can be treated as a one-dimensional, time-dependent process with distance $\gamma$ measured radially from the CC. The micrograph is modelled by a radially symmetric 2D Cartesian plane where the cells are represented by compartments taking up the area $V_{{{\text{in}}}}$ and the outside $V_{{{\text{out}}}}$, and the total sum $V = V_{{{\text{in}}}} + V_{{{\text{out}}}}$. Since the micrograph is three dimensional but thin, we label its depth to be $h_{0} = 2 \mu m;$ the length of an average *E. coli* cell. The fraction $\beta = V_{{{\text{in}}}} /V$ labels how densely the micrograph is populated by cells (in our case, $\beta = 0.41$). The modified Goldman and continuity equations in dimensionless time ($\tilde{t} = \frac{t}{\tau }$) are^24^:

12 $$\left\{ {\begin{array}{*{20}c} {\frac{{\partial c_{1} }}{\partial t} = \alpha P\left( {\gamma ,t} \right)\left( {\frac{{c_{2} \alpha_{m} - c_{1} }}{{1 - \alpha_{m} }}} \right)} \\ {\frac{{\partial c_{2} }}{\partial t} = \left( {\frac{\beta - 1}{\beta }} \right)\frac{{\partial c_{1} }}{\partial t}} \\ \end{array} } \right.$$

Here, $\alpha = p_{0} \tau \phi /\kappa$ is the scaling factor, $p_{0}$ is characteristic permeability, time of observation $\tau = 600$ s, the dimensionless transmembrane potential $\phi = 2e_{0} \Delta \Phi_{m} /k_{B} T$ and $\alpha_{m} = {\text{exp}}\left( { - \phi } \right).$ Additionally, $e_{0}$ is the elementary charge, + 2 is the valence of a single PI ion, $\Delta \Phi_{m}$ is the transmembrane electrical potential (125 mV)^25^, $k_{B}$ the Boltzmann constant, $T$ absolute temperature and $c_{1} \left( {\gamma ,t} \right)$ and $c_{2} \left( {\gamma ,t} \right)$ are the dimensionless concentrations of PI outside and inside the bacterial cells, respectively. $\kappa = 8.68 \cdot 10^{5}\kappa = 8.68 \cdot 10^{5}$ m marks the ratio between volume not occupied by cells and their total surface area $\frac{{V_{{{\text{out}}}} }}{{N_{{\text{e}}} A_{{\text{e}}} }} = ar_{{\text{e}}} \left( {\frac{1}{{\pi r_{e}^{2} }} - \rho } \right)/\left( {2\rho \left( {r_{{\text{e}}} + a} \right)} \right)$. Here, $N_{{\text{e}}}$ is the number of *E. coli* cells within the relevant observation area and $A_{{\text{e}}}$ the surface area of an average cell modeled as a cylinder with length $a = 2 \mu m$ and radius $r_{{\text{e}}} = 0.5 \mu m$. The measured planar density of cells was $\rho = 0.275/\mu m^{2}$. Note that hereafter, the tilde is omitted and $t$ is dimensionless. Since PI can be either inside or outside the cells, the normalizing condition $c_{1} \left( {\gamma ,t} \right) + c_{2} \left( {\gamma ,t} \right) = 1$. Because PI is added to the solution prior to microbubble collapse, the initial conditions are

13 $$c_{1} \left( {\gamma ,0} \right) = 1,c_{2} \left( {\gamma ,0} \right) = 0$$

We may solve the Goldman equation by considering the nominal size of the constants. The dimensionless transmembrane potential is $\phi$ = 11.57 for $T = 300$ K, which means that $\alpha_{m} = {\text{exp}}\left( { - \phi } \right) \approx 0$. This leads us to rewrite Eq. (12):

14 $$\frac{{\partial c_{1} }}{\partial t} = - \alpha P\left( {\gamma ,t} \right)c_{1} .$$

For a point at constant $\gamma$, this equation may be separated and integrated on both sides:

15 $$\mathop \smallint \limits_{{c_{1} \left( {t = 0} \right)}}^{{c_{1} \left( t \right)}} \frac{{\partial c_{1} }}{{c_{1} }} = - \mathop \smallint \limits_{0}^{t} \alpha P\left( {\gamma ,t} \right)\partial t.$$

We know that permeability can be factored into its spatial and temporal parts (Eq. (9)): $P\left( {\gamma ,t} \right) = \frac{{kk_{1} }}{{p_{0} }}I_{final} \left( \gamma \right)e^{ - \theta t}$. Here, $\theta = k\tau$ is the dimensionless constant. Therefore, we can factor out the parts not dependent on time

16 $${\text{ln}}\left( {\frac{{c_{1} \left( t \right)}}{{c_{1} \left( 0 \right)}}} \right) = \frac{{ - \alpha kk_{1} }}{{p_{0} }}I_{{{\text{final}}}} \left( \gamma \right)\mathop \smallint \limits_{0}^{t} e^{ - \theta t} dt$$

The initial condition $c_{1} \left( 0 \right) = 1$ (Eq. (13)), while the right-hand side is integrated to give

17 $${\text{ln}}(c_{1} \left( t \right)) = \frac{{\alpha kk_{1} I_{{{\text{final}}}} \left( \gamma \right)}}{{\theta p_{0} }}\left( {e^{ - \theta t} - 1} \right)$$

The constants on the right side simplify to $\phi k_{1} /\kappa$.aking another exponent gives the final expression for $c_{1} \left( t \right)$:

18 $$c_{1} \left( t \right) = {\text{exp}}\left[ {\frac{{\phi k_{1} }}{\kappa }I_{{{\text{final}}}} \left( \gamma \right)\left( {e^{ - \theta t} - 1} \right)} \right]$$

Because of PI particle conservation, $c_{2} \left( {\gamma ,t} \right) = 1 - c_{1} \left( {\gamma ,t} \right)$. With this, we get an expression for $c_{2} \left( {\gamma ,t} \right)$:

19 $$c_{2} \left( {\gamma ,t} \right) = 1 - {\text{exp}}\left[ {\frac{{\phi k_{1} }}{\kappa }I_{{{\text{final}}}} \left( \gamma \right)\left( {e^{ - \theta t} - 1} \right)} \right]$$

We examine the constants $\frac{{\phi k_{1} }}{\kappa }$, which simplify to $\frac{{\phi k_{1} }}{\kappa } = 2.72\cdot10^{ - 11} /AU$. This is a small factor, so we can expand the exponent into a series:

20 $$c_{2} \left( {\gamma ,t} \right) = \frac{{\phi k_{1} }}{\kappa }I_{{{\text{final}}}} \left( \gamma \right)\left( {1 - e^{ - \theta t} } \right)$$

We notice that this is the same dependency as $I\left( {\gamma ,t} \right)$ (Eq. (7)), which confirms our initial hypothesis that PI concentration inside the cells is in direct proportion to the measured fluorescence signal. We can relate both quantities by units, so $I = c_{2} c_{0} k_{2}$, where $c_{0}$ is the initial concentration of PI in the solution (60 nM) and $k_{2}$ is the conversion factor between concentration and fluorescence intensity with units $k_{2} = 3.4\cdot10^{6}$ AU m^3^/mol. Comparing Eq. (20) with Eq. (7), we find that

21 $$\frac{{\phi k_{1} k_{2} c_{0} }}{\kappa } = 1$$

Since $\kappa = \frac{{V_{{{\text{out}}}} }}{{N_{{\text{e}}} A_{{\text{e}}} }}$ and $\phi = ze_{0} \Delta \Phi_{m} /k_{B} T$, where $z$ is the valence of the fluorescent dye used, we derive, as a side note, an equation that connects the transmembrane potential of the studied cells with the phenomenological constants $k_{1}$ and $k_{2}$:

22 $$\Delta \Phi_{m} = \frac{{V_{{{\text{out}}}} k_{B} T}}{{ze_{0} N_{{\text{e}}} A_{{\text{e}}} c_{0} k_{1} k_{2} }}$$

The transmembrane potential depends on the uptake kinetics of the fluorescent dye and is inversely proportional to the initial concentration of the dye incubated together with the cells. Also important is the shape and density of cells enclosed in volume $V_{{{\text{out}}}}$, since a larger surface-to-volume ratio will expose more surface area to dye on the outside.

### Comparison of experimental and modeling results

Model predictions show good agreement with the experimental observations (Fig. 4). Figure 4A illustrates the spatiotemporal experimentally observed progression of dye uptake. Radial spreading of the uptake from the CC is evident from the increasing number of stained cells over time. Figure 4B compares the spatial variation of final fluorescence intensity, $I_{{{\text{final}}}} \left( {\gamma , \tau } \right)$, as observed experimentally and predicted by the model. Overall agreement was strong, though discrepancies were greatest at short distances from the CC. This may be due to the blast radius displacing cells away from the CC, leading to an underestimation of intensity in that region.

Figure 4C shows the predicted damage ratio, $S_{{\text{p}}}$ (Eq. (11))*,* representing the fraction of damaged cell membranes area relative to the total membrane area as a function of distance and time. Figure 4D summarizes the key derived parameters, including the damage ratio immediately after the cavitation ($t_{{0}} = 0$ s), the final damage ratio ($t_{{{600}}} = 600$ s), and $I_{{{\text{final}}}}$ at representative $\gamma$ values.

The results indicate that cells close to the CC ($\gamma$ ≈ 0.14) were predicted to reach a maximum fluorescence intensity exceeding 2500 AU, with a 100% damage ratio at t = 0 s. In contrast, cells near the projected maximum bubble radius ($\gamma$ ≈ 1) exhibited a maximum fluorescence intensity of approximately 500 AU, an initial damage ratio of 33.1%, and a final damage ratio of 1.7%.

## Discussion

The dynamics of a single cavitation microbubble interaction with bacterial cells are not well understood. The results of this study present spatial and temporal data about the propagation of bacterial cell poration from a single cavitation event. We have combined ultra-high-speed imaging with time-resolved fluorescence analysis to capture bacterial membrane responses induced by high-frequency mechanical perturbation of the membrane. The developed numerical model connects experimental data with propidium iodide (PI) uptake dynamics and provides temporal resolution not available by the experiment. Such an approach provides a unique insight into the spatial and temporal relationship between a single cavitation event and consequential molecular transport of PI across damaged bacterial cell membranes, which is implicitly measured with fluorescence signal distribution. The selected fluorescence marker (PI) is a small and positively charged molecule that can pass through the outer membrane of *E. coli* via porin channels such as OmpF and OmpC, with reported transport rates on the order of 10⁻^2^ s⁻^1^^26,27^. However, the cytoplasmic membrane poses a more significant barrier, with passive transport occurring much more slowly, often requiring external perturbation such as sonoporation or electroporation to facilitate the passage^27–29^.

The spatial extent of the damage on a layer of cells inflicted by the single bubble cavitation event was observed before ^30–35^, but the spatiotemporal analysis has not been attempted. Recent single-bubble experiments showed that bacterial cells are affected by the cavitation bubble only in its immediate vicinity^17^. Known mechanisms for bacterial inactivation by cavitation bubbles are jet formation, microstreaming and shock waves or ROI formation, which could induce membrane permeabilization in presented case^36^. Bubble collapse near a rigid surface produces a jet that generates high mechanical load towards the surface, where cells are detached or damaged^17,30–35^. At supplementary video 1 one could observe that cell detachment occurs in the collapsing phase of the cavitating bubble, which further confirms jetting as the main mechanism of the attached cell damage^17^. The distance of the bubble from the surface (i.e. attached cells) can also have various effects on bubble dynamics and cells alike^32,37^. In the presented experimental setup, the nucleation bubble was close to the surface (approx. 1–4 µm above the surface). This results in fast needle jet formation^17,38–40^, which we were not able to observe with the current visualization setup.

Most of the previous research followed the effect of cavitation only at a single time point after cavitation. Our presented research focused on damage induced by a cavitation bubble on a larger scale. By examining PI uptake dynamics for 5565 *E. coli* cells, we found that the cavitation bubble has a delay effect away from the cavitation center. Most fascinating is the observed discrepancy between the observed PI uptake timescale of minutes versus the microsecond timescales of the mechanical shear stress induced by cavitation event. This suggests a significant temporal decoupling between mechanical stress application and transport of PI into the cells. The evidence of cell damage stems from the fact that PI is a molecule that cannot transverse the cellular membrane and induce a fluorescent signal without membrane pores. While pore formation under mechanically stress occurs rapidly^41^, the resulting membrane damage can persist from milliseconds to several minutes, depending on the pore size and membrane properties^42,43^. This observation is consistent with literature showing that pore stability and resealing dynamics depend not only on mechanical parameters but also on intrinsic cellular factors such as membrane tension and cytoskeletal anchorage^42^. Following the membrane disruption in bacteria, for example, by electroporation or antimicrobial peptides, the uptake of small molecules into the cytoplasm often exhibits a time-dependent exponential decay^44,45^. A similar exponential response of fluorescence intensity could be observed also in our experimental data of the single bacterial fluorescence intensity. This reflects the transient and time dependent nature of membrane permeability; immediately after poration, multiple membrane defects allow rapid influx of PI, but as the membrane begins to reseal, permeability decreases^46^.

To quantitatively describe PI uptake, we employed a modified Goldman equation to model ion and molecular flux across the bacterial membrane. Our observation of increased fluorescence over time and eventual saturation aligns well with the passive transport kinetics through transient pores as observed also in previous studies^26–28,47^. Moreover, the similarity of our observed time constant, on the order of minutes (3.4 min), to previously reported uptake plateaus in electroporated or mechanically stressed cells (e.g., 2.5 min in Jurkat cells) supports the interpretation that our results reflect diffusion-limited PI uptake through long-lived membrane pores^48,49^. Importantly, the transport induced by cavitation based on numerical model appears to be non-specific and bidirectional, consistent with established sonoporation mechanisms^50–53^. Studies using real-time tracking of molecules like calcein and FITC-dextran show similar behaviors, where smaller molecules diffuse freely while larger ones experience reduced transport into the cell^54^.

By combining previous work on cavitation bubble dynamics and threshold hydrodynamic forces required to stain *E. coli* cells^17^, with the presented uptake model, it can be estimated that hydrodynamic forces of approximately 1.2 µN are expected at a distance of 9 µm. This level of force could result in an initial cell damage ratio of about 44.1% at the time of microbubble collapse, which decreases to 2.3% after 600 s. At larger distances, around 30 µm, the hydrodynamic force is estimated to be 52 nN, potentially resulting in an initial damage ratio of 33.1%, which declines to approximately 1.7% by the end of the observation period. These estimations underscore the relationship between the magnitude of mechanical stress and the extent of membrane permeabilization and resealing, thereby contributing to a mechanistic understanding of cavitation-induced molecular transport.

While it is not possible to directly observe the opening and closing of individual pores in real time, our model infers this transient pore behavior as necessary to match the experimental fluorescence data. Micrographs alone cannot confirm pore resealing, but the model shows that pores must close over time to account for the saturation in PI uptake. Although cells in the center of the cavitation bubble are detached or lysed, consistent with jetting-induced mechanical stresses^17,30,32–35^, many cells in the peripheral region remain structurally intact but transiently permeabilized. This is consistent with sonoporation literature, where cavitation creates distinct zones of effect: irreversible cell lysis near the bubble center and reversible poration farther away^55,56^. The resealing kinetics observed here suggest that at least a fraction of PI-positive cells likely survive after membrane repair, as seen in electroporation and sonoporation studies where 60–90% of cells with pores recover once pores reseal^57–59^. The coupling between the macro-scale model and micro-scale membrane behavior is a novel and important insight. The model successfully links fluorescence intensity to the underlying biophysical processes, demonstrating that PI uptake follows first-order kinetics with a unified time constant on the order of minutes. Notably, the final fluorescence intensity exhibits an inverse dependence on the radial distance from the cavitation event, suggesting that mechanical energy from cavitation dissipates in a 2D-like manner. The derived damage ratio shows an initial spike in membrane disruption followed by an exponential decay as pores reseal with time. However, some limitations exist. Within the paper there is presented only one experimental case of the PI uptake. Although the repeatability of the effect of single microbubble on the *E. coli* cells has been demonstrated^17^, the uptake dynamics of individual cells can vary slightly across experiments and are not fully cross correlated. When comparing different experimental data, qualitative agreement is observed in the general trend, although quantitative discrepancies exist (Supplementary Fig. 3). These differences may arise from variability in bubble dynamics, bacterial cell responses, or other unknown factors. Furthermore, the assumption of purely radial symmetry may oversimplify real-world scenarios where bubble collapse can be asymmetric. Additionally, the model treats membrane permeability as homogeneous, whereas real bacterial membranes may exhibit localized variations in pore distribution. Future work could focus on improving experimental setup to enhance spatial and temporal resolution, as well as incorporating stochastic pore dynamics or 3D effects to refine predictions. Overall, this model provides a robust framework for understanding cavitation-induced membrane damage and PI uptake, with implications for biomedical applications such as drug and molecule delivery. Together, these results deepen our mechanistic understanding of cavitation-induced membrane poration and molecular transport in bacteria. By bridging high-resolution imaging with quantitative modeling, we provide a foundation for tuning cavitation parameters in biomedical applications ranging from drug delivery to targeted antimicrobial therapies. Future work integrating asymmetric bubble dynamics, heterogeneous membrane responses, and larger biomolecular cargoes will further enhance the predictive power of this approach.

## Conclusion

In conclusion, our study demonstrates that a single microscale cavitation event can induce localized, transient membrane permeabilization in *Escherichia coli (E. coli)* with high spatial and temporal precision. Using high-speed imaging and propidium iodide (PI) fluorescence tracking, we monitored the dynamic response of over 5500 individual cells and observed that membrane damage and PI uptake decreased symmetrically with increasing distance from the cavitation center. A modified Goldman model accurately described the PI uptake kinetics and indicated that membrane permeability declines exponentially over time, suggesting a spontaneous pore-resealing process. Importantly, the model enabled estimation of parameters that are difficult to access experimentally, such as the extent of membrane damage over time and distance. Our findings are consistent with previous sonoporation studies and provide new insights into the mechanisms of cavitation-induced molecular transport, offering a framework for optimizing single-bubble cavitation in applications such as targeted drug delivery, microbial control, and biofilm disruption.

## Supplementary Information

Below is the link to the electronic supplementary material.

Supplementary Material 1

Supplementary Material 2

Supplementary Material 3

Supplementary Material 4

## Data Availability

The datasets generated and/or analyzed during the current study are available from the corresponding author on reasonable request.
