# Supplementary material for: Spatiotemporal analysis of Escherichia coli membrane permeabilization and uptake kinetics induced by a single microbubble cavitation event
Source: Sci Rep. 2025 Nov 28;15:42593. doi: 10.1038/s41598-025-26554-4 (PMC12663178; doi:10.1038/s41598-025-26554-4)
Supplement: Supplementary file 1 — Supplementary Material 1 [file 41598_2025_26554_MOESM1_ESM.docx]

Supplementary information for

**Spatiotemporal analysis of *Escherichia coli* membrane permeabilization and uptake kinetics induced by a single microbubble cavitation event**

Mitja Drab^a,b^, Aleš Iglič^a^, David Stopar^c^, Žiga Pandur^b^*

*^a^ Faculty of Electrical Engineering, University of Ljubljana, Tržaška cesta 25, 1000 Ljubljana, Slovenia
^b^ Faculty of Mechanical Engineering, University of Ljubljana, Aškerčeva 6, 1000 Ljubljana, Slovenia
^c^ Biotechnical Faculty, University of Ljubljana, Jamnikarjeva 101, 1000 Ljubljana, Slovenia*

* Corresponding author: <ziga.pandur@fs.uni-lj.si>

1. **Supplementary data**

Supplementary video 1:

Cavitation microbubble dynamics captured with ultra-high speed camera. A small initial nucleus bubble in the center of the FOV (not visible) expands and collapses, while forming a characteristic jet at the collapse phase. Maximum bubble radius was measured 29.7 µm. Scale bar 20 µm.

Supplementary video 2:

Staining of bacterial cells after microbubble cavitation. In the supplementary video it is shown full time lapse video with 0.5s between the frames. In the video, one could observe also some floating cells in the FOV that were also stained with PI and were probably detached from the center of the bubble collapse. This is most notable at the longer incubation periods, where the unattached cells slowly moved to the right side of FOV where one could observe eccentric pattern of the stained cells.

Supplementary video 3:

Animation of numerical analysis for fluorescence intensity dynamics through space (distance γ) through time. X axis represents distance γ, where bubble center is positioned at γ = 3. At the t = 0, the fluorescence is at 0, right after the cavitation event, the fluorescence rapidly starts to increase around the bubble center (γ = 3).

Supplementary Figure 1:

The residual analysis for Figure 2A together with its coefficient of determination (R²) and RMSE value.


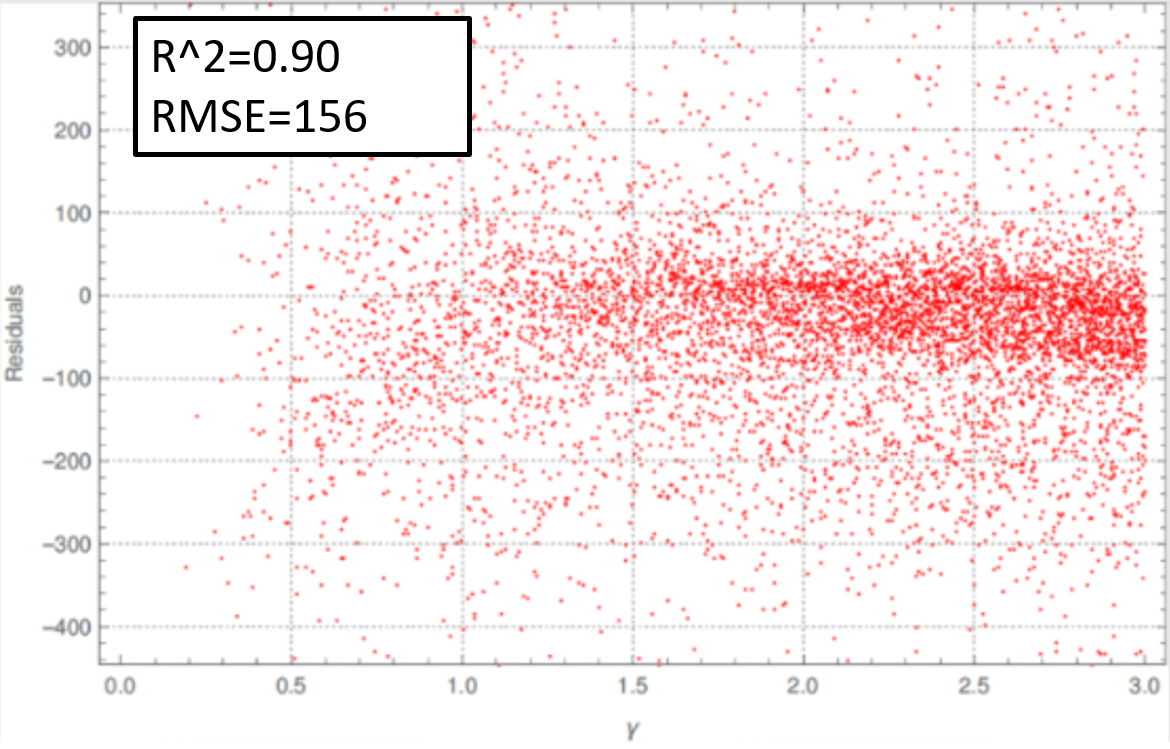


The residual analysis for Figure 2B together with its coefficient of determination (R²) and RMSE value.

Supplementary Figure 2:


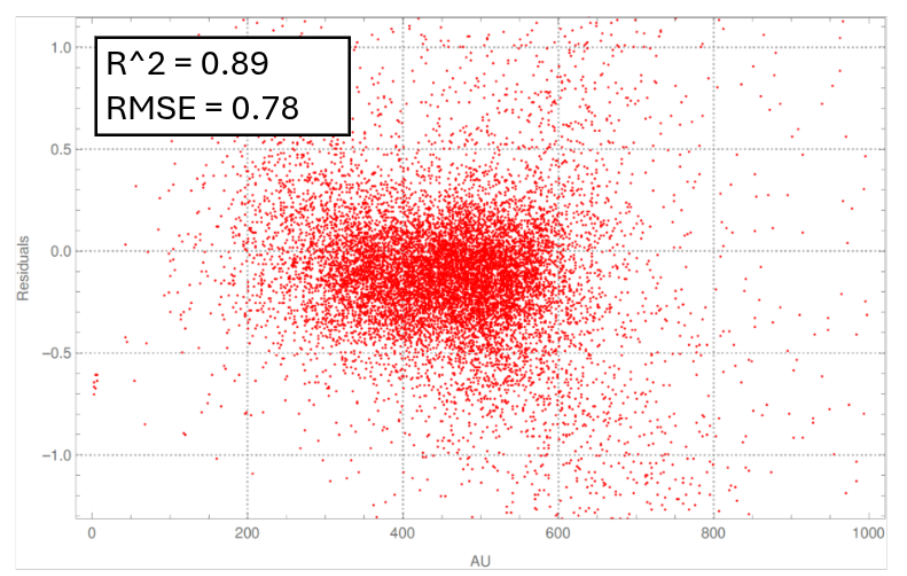


The residual analysis for Figure 2B together with its coefficient of determination (R²) and RMSE value.

Here, the R^2 and RMSE and residuals were calculated according to the method described below: The coefficient of determination R^2 is calculated as R^2 = 1 – (SSres / SStot), where SSres = sum((yi – ŷi)^2) is the residual sum of squares, yi are the observed values, and ŷi are the predicted values from the model. SStot = sum((yi – ȳ)^2) is the total sum of squares, with ȳ being the mean of the observed values.

The residuals are simply the differences between observed and predicted values, that is residuals = yi – ŷi for each data point. By plotting these residuals against the independent variable or against the fitted values, one can visually assess whether there is any systematic pattern left unexplained by the model. The perfect case is where the residuals are all scattered homogenously around the axis 0, which is roughly the case in our analysis.

The root mean squared error (RMSE) is calculated as RMSE = sqrt(mean((yi – ŷi)^2)), which is the square root of the average squared residual. RMSE gives the typical size of the error in the same units as the dependent variable, making it easy to interpret relative to the scale of the data.

Supplementary Figure 3:


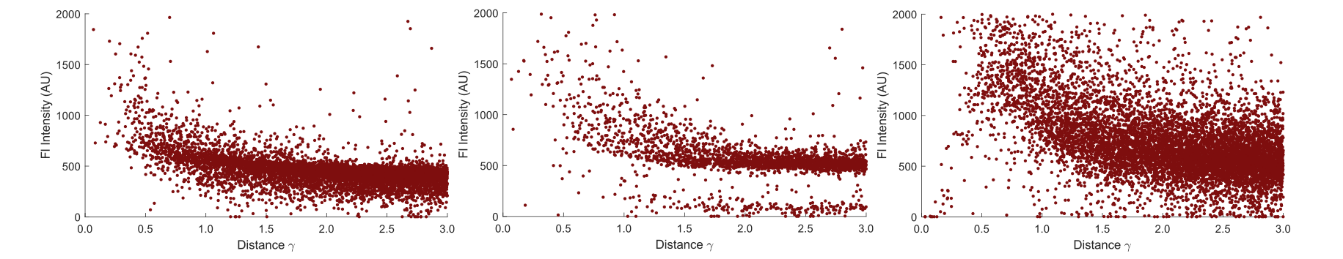


Additional experimental data showing single-cell fluorescence intensity as a function of γ-distance at 600 s after the cavitation bubble event. The left panel presents the analyzed data included in the manuscript, while the middle and right panels show results from additional experiments. In all cases, the overall increasing trend of PI uptake with γ-distance is qualitatively comparable.
